# An invasive granular cell tumor originating from the left rectus abdominis muscle: a case report

**DOI:** 10.3389/fsurg.2026.1808303

**Published:** 2026-03-20

**Authors:** Tao Huang, Hanbo Liu, Tonggang Yu, Shangkun Li

**Affiliations:** 1 Department of General Surgery, Tianjin Xiqing Hospital, Tianjin, China; 2 Department of Radiology, Tianjin Xiqing Hospital, Tianjin, China

**Keywords:** abdominal wall, abdominal wall reconstruction, case report, granular cell tumor, rectus abdominis, surgery

## Abstract

**Background:**

Granular cell tumors (GCTs) are rare soft tissue neoplasms, with invasive forms being exceptionally uncommon in the abdominal wall. This case highlights the diagnostic and therapeutic challenges of managing an invasive GCT in an atypical location.

**Case presentation:**

A 50-year-old male presented with a painless, enlarging mass in the left rectus abdominis, with no history of trauma or systemic symptoms. Imaging revealed an exophytic mass with ill-defined borders. Histopathological examination confirmed an invasive granular cell tumor. The patient underwent complete surgical resection with partial muscle excision, followed by abdominal wall reconstruction using mesh to prevent hernia formation.

**Conclusion:**

Invasive GCTs can occur in the abdominal wall and require complete resection with meticulous reconstruction to ensure both oncological safety and functional integrity. This case underscores the importance of considering GCTs in the differential diagnosis of abdominal wall masses.

## 1 Introduction

Granular cell tumors (GCTs) are uncommon soft tissue tumors with an incompletely understood histogenesis. While most GCTs are benign, typically presenting as painless, slowly growing masses in the oral cavity, skin, or subcutaneous tissues, malignant or invasive variants account for less than 2% of cases. The occurrence of GCTs within the skeletal muscle of the abdominal wall is exceptionally rare, with only a handful of cases reported in the literature. They lack specific diagnostic features and are often misdiagnosed preoperatively as common superficial subcutaneous lesions, such as fibromas or lipomas, posing significant challenges for differential diagnosis. For the few cases of invasive GCTs, contrast-enhanced CT may offer certain diagnostic value. However, due to the clinical perception that superficial masses are usually benign, the high cost of CT scans, iodine contrast allergies in some patients, and the fact that many such lesions are managed as outpatient surgeries, contrast-enhanced CT is often not performed. As a result, the diagnosis is frequently confirmed only postoperatively through histopathological examination. We present a case of an invasive GCT arising from the left rectus abdominis muscle, a location that poses unique diagnostic and therapeutic challenges due to the functional anatomy of the abdominal wall.

## 2 Case presentation

A 50-year-old male with no significant past medical history presented to our general surgery department with a chief complaint of a painless mass on his left abdominal wall that had been present for 6 months. The mass had gradually increased in size from that of a soybean to a quail egg. Physical examination revealed a firm, non-tender, poorly mobile mass approximately 3 cm in diameter in the left mid-abdomen. Abdominal CT plain scan showed an exophytic mass on the left abdominal wall with an indistinct boundary with the rectus abdominis muscle (Supplementary Figures 1A, B). Color Doppler ultrasound confirmed a solid mass within the muscle layer (Supplementary Figure 1C).

The patient underwent surgical resection under general anesthesia. Intraoperatively, the tumor was found to be invasive, involving the anterior sheath and medial muscle bundle of the rectus abdominis. The mass was excised *en bloc* with a margin of healthy muscle tissue. Due to the defect created by the resection, an 8cm × 12 cm polypropylene mesh was placed in a retromuscular position to reconstruct the abdominal wall and prevent hernia formation.

Histopathological examination revealed sheets of polygonal cells with abundant eosinophilic granular cytoplasm, consistent with a granular cell tumor. Immunohistochemical staining was positive for S-100 and CD68, confirming the diagnosis. The tumor exhibited infiltrative growth patterns, leading to a diagnosis of invasive granular cell tumor (Supplementary Figure 1D).

The patient's postoperative course was uneventful. He was discharged after wound healing and was followed up regularly. A CT plain scan at 1-year post-surgery showed no evidence of recurrence, with only a reticular density shadow in the surgical area (Supplementary Figure 1E).

## 3 Discussion

Granular cell tumors (GCTs) are relatively rare tumors with unknown histogenesis. There is some controversy over its histogenesis, but based on immunohistochemistry, electron microscopy and typical lesions located in nerves, it is believed to be derived from nerve sheath cells and is a disease that leads to the aggregation of lysosomes in the cytoplasm, mainly seen in nerve sheath cells and other types of cells. A diagnosis of GCTs will only be made when the entire lesion shows granular changes. As a rare tumor (1), GCTs can occur in any part of the body, mainly in the oral cavity, chest wall and arms (2–4). There are also less common sites, such as the throat, digestive tract, spine, anogenital area, etc (5, 6). It can also occur on the skin or in the upper third of the right rectus abdominis muscle of the abdominal wall.

GCTs are uncommon soft tissue neoplasms of Schwann cell origin (7), typically presenting as benign, slow-growing masses. However, the invasive variant, as observed in our case, poses significant diagnostic and therapeutic challenges, particularly when located in the abdominal wall.

The initial diagnostic challenge in our patient stemmed from the tumor's aggressive imaging appearance. Unlike the typical well-circumscribed benign GCTs, our case exhibited ill-defined borders and an exophytic growth pattern on imaging, which initially raised suspicion for a malignant sarcoma or desmoid tumor. This aligns with the literature, which reports that invasive GCTs can mimic malignancies due to their infiltrative nature (8). The definitive diagnosis was only established through histopathological examination, highlighting the indispensable role of biopsy in ambiguous soft tissue lesions.

The cornerstone of treatment for invasive GCTs is complete surgical excision with negative margins. In our case, the tumor's location within the rectus abdominis muscle necessitated a partial muscle excision. This created a significant defect that required reconstruction to prevent abdominal wall hernia. The use of a synthetic mesh for reinforcement was a critical step in our management (9). This approach not only ensured oncological safety by allowing wide local excision but also preserved the functional integrity of the abdominal wall (8), a balance that is crucial in truncal soft tissue surgery.

This case underscores the importance of considering invasive GCTs in the differential diagnosis of abdominal wall masses, even in the absence of typical features. The 1-year follow-up without recurrence in our patient supports the efficacy of the R0 resection strategy. However, given the potential for local recurrence in invasive variants, long-term surveillance remains mandatory.

The diagnosis of benign or malignant granular cell tumors can be guided by the Fanburg-Smith criteria (10), which involve evaluating six histological features: necrosis, spindling, vesicular nuclei with large nucleoli, increased mitotic activity (>2 mitoses/10 high-power fields at 200× magnification), high nuclear-to-cytoplasmic ratio, and pleomorphism. Tumors meeting three or more of these criteria are classified as histologically malignant. In the broader context of soft tissue pathology, the presentation of GCTs with invasive features in the abdominal wall is exceptionally rare. A review of the literature reveals that while GCTs are most commonly found in the head and neck region (11–13), truncal involvement is less frequent, for instance, it may occur in the right rectus abdominis muscle (7, 14, 15), and invasive behavior in this location is a notable deviation from the norm (16). This case adds to the limited body of evidence suggesting that anatomical location may influence the biological behavior of these tumors. Furthermore, the differential diagnosis for an infiltrative abdominal wall mass must include entities such as desmoid tumors, which are locally aggressive but non-metastatic, and undifferentiated pleomorphic sarcoma. The key distinguishing feature in our case was the histopathological hallmark of granular cells with abundant eosinophilic cytoplasm and positive immunohistochemical staining for S-100 protein (1) and CD68 (15), which is characteristic of GCTs and helps differentiate them from other spindle cell neoplasms. Pathological diagnosis of this case: Granular cell tumor of the abdominal wall, exhibiting locally invasive growth with malignant potential in the muscular layer. Therefore, we consider it to possess invasive characteristics.

The decision to proceed with mesh reconstruction after tumor resection was guided by the principles of abdominal wall oncology. While primary closure might be sufficient for small defects, the size and depth of the resection in this case mandated reinforcement to prevent incisional hernia and maintain core stability. Prognostically, invasive GCTs occupy a gray zone between benign and malignant. Although they lack the high metastatic potential of true malignant GCTs, their infiltrative growth pattern confers a higher risk of local recurrence compared to their benign counterparts. Therefore, the surgical goal must be complete macroscopic and microscopic removal, as residual disease is the primary driver of recurrence.

In summary, this case illustrates that invasive GCTs can present in atypical locations like the abdominal wall. A multidisciplinary approach involving radiology, pathology, and surgery is essential for accurate diagnosis and optimal management, with a focus on achieving both oncological clearance and functional restoration.

## 4 Conclusion

This case report underscores that invasive GCTs can occur in the abdominal wall musculature. Surgical resection with clear margins is the treatment of choice. Given the anatomical constraints, concomitant abdominal wall reconstruction with mesh should be considered to ensure both curative intent and functional recovery.

## 5 Patient perspective

The patient was initially anxious about the growing mass. After surgery and explanation of the diagnosis, he was relieved and satisfied with the cosmetic and functional outcome. He consented to the publication of this case to help other patients with similar conditions.

## Data Availability

The original contributions presented in the study are included in the article/Supplementary Material, further inquiries can be directed to the corresponding author.
