# Supplementary material for: An invasive granular cell tumor originating from the left rectus abdominis muscle: a case report
Source: Front Surg. 2026 Mar 20;13:1808303. doi: 10.3389/fsurg.2026.1808303 (PMC13047077; doi:10.3389/fsurg.2026.1808303)
Supplement: Supplementary file 1 [file Supplementaryfile1.docx]

Supplementary Material

# Supplementary Figures


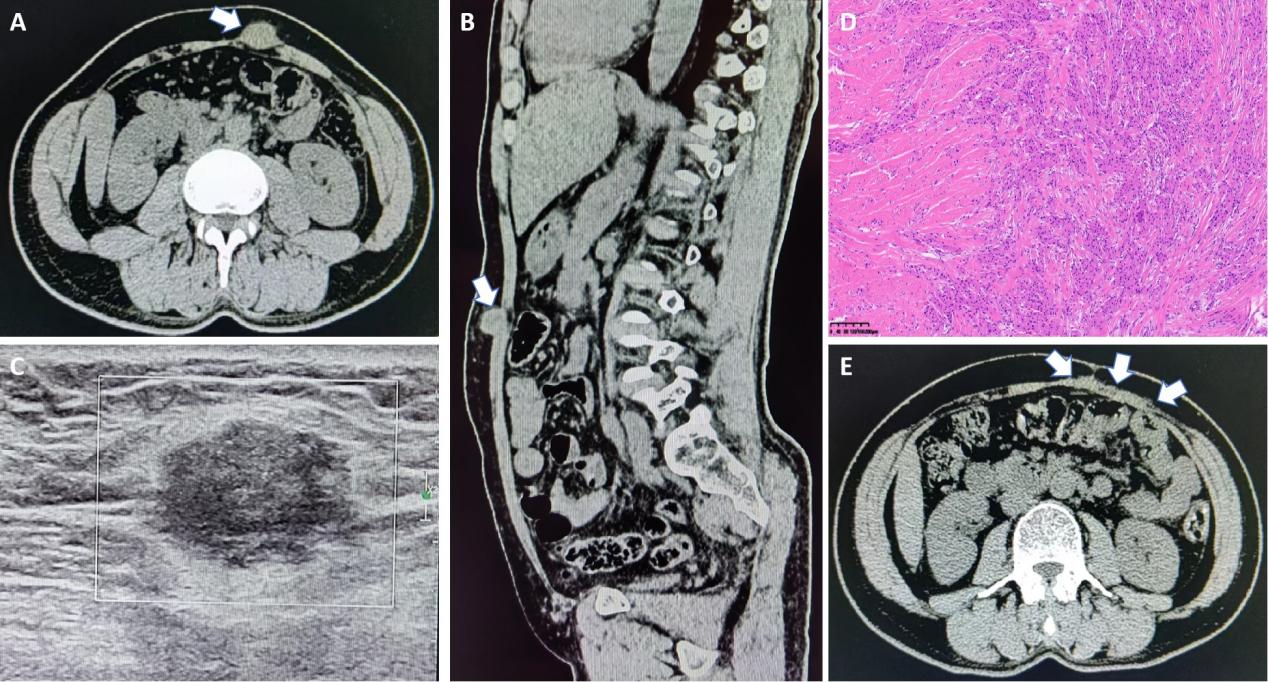


**Supplementary Figure 1.**

（A）Transverse CT image of granular cell tumor (white arrow).

（B）Sagittal CT image of granular cell tumor (white arrow).

（C）Color Doppler of abdominal wall mass.

（D）Postoperative pathological histological examination(H&E stain,×200).

（E）Transverse CT image of the operative area one year after surgery(white arrow).The exophytic mass on the left abdominal wall did not reappear, and there was a reticular density shadow in the surgical area.
